# Perioperative and long-term outcomes of laparoscopic liver resection for combined hepatocellular carcinoma and cholangiocarcinoma versus intrahepatic cholangiocarcinoma: A propensity score matching analysis

**DOI:** 10.1371/journal.pone.0328104

**Published:** 2025-08-18

**Authors:** Chengfei Du, Hanyu Wang, Wenli Cao, Zichen Yu, Junwei Liu, Jie Liu, Liming Jin, Yunyun Feng, Fangqiang Wei

**Affiliations:** 1 Department of General Surgery, Cancer center, Division of Hepatobiliary and Pancreatic Surgery, Zhejiang Provincial People’s Hospital, Affiliated People’s Hospital, Hangzhou Medical College, Hangzhou, Zhejiang Province, China; 2 Second Clinical Medical College, Zhejiang Chinese Medical University, Hangzhou, Zhejiang Province, China; 3 Department of Public Health, Hangzhou Medical College, Hangzhou, Zhejiang Province, China; PLOS: Public Library of Science, UNITED KINGDOM OF GREAT BRITAIN AND NORTHERN IRELAND

## Abstract

**Background:**

Laparoscopic liver resection (LLR) has been increasingly used to treat intrahepatic cholangiocarcinoma (ICC), yet the role of LLR on combined hepatocellular carcinoma and cholangiocarcinoma (cHCC-CC) remains unclear. The purpose of this study was to compare the perioperative efficacy and long-term prognosis of LLR for cHCC-CC and ICC through the use of propensity score matching (PSM) analysis.

**Methods:**

Clinicopathologic, perioperative, and survival data of patients with cHCC-CC and ICC who underwent LLR from November 2018 to May 2023 at our institution were retrospectively collected. The two groups were further analyzed using 1:1 PSM to compare perioperative outcomes and long-term prognosis.

**Results:**

A total of 115 patients who underwent LLR for either eHCC-CC or ICC were ultimately included in the study. Among them, there were 24 cases in the cHCC-CC group and 91 cases in the ICC group. After PSM, the cHCC-CC group exhibited a significantly higher prevalence of preoperative elevated AFP levels (45.8% vs. 0, P < 0.001) compared with the ICC group. The two groups were comparable in terms of perioperative data. After a median follow-up of 34 months, there were no significant difference in 1-year OS (92% vs. 88%), 2-year OS (62% vs. 70%), 3-year OS (49% vs. 59%), 1-year RFS (46% vs. 58%), 2-year RFS (29% vs. 54%), 3-year RFS (29% vs. 42%) between the cHCC-CC and ICC groups (all P > 0.05).

**Conclusions:**

The perioperative outcomes and long-term prognosis of LLR for patients with cHCC-CC are comparable to those observed in patients with ICC.

## 1. Introduction

Combined hepatocellular carcinoma and cholangiocarcinoma (cHCC-CC) is a rare primary hepatic malignancy characterized by morphologic differentiation of hepatocytes and biliary epithelial cells, accounting for approximately 0.4% to 14.2% of primary liver cancers [1–3]. It has been reported that cHCC-CC and intrahepatic cholangiocarcinoma (ICC) exhibit similar clinical features and histopathologic characteristics, indicating a genetic proximity between the two malignancies [4–6]. cHCC-CC is typically characterized by high malignancy and a rapid progression. While hepatic resection is currently an effective treatment option for cHCC-CC, its long-term prognosis, unfortunately, remains unsatisfactory, similar to that of ICC [6]. More than half of the patients experience recurrence after surgery, and the 5-year survival rate is less than one-third [7,8].

In recent years, LLR has gained widespread application in the treatment of various liver malignancies due to its distinct advantages, including minimal intraoperative bleeding, a high R0 resection rate, and a low incidence of postoperative complications [9,10]. An increasing number of studies have demonstrated that LLR is a safe and effective treatment option for most patients with ICC [11–13]. However, there have been few studies reported on the use of laparoscopic techniques for treating cHCC-CC. To the best of our knowledge, there is no record of the difference in prognosis between the laparoscopic treatment of cHCC-CC and ICC. The aim of this study was to compare the perioperative efficacy and long-term prognosis of LLR for the treatment of cHCC-CC and ICC through a propensity score matching (PSM) analysis.

## 2. Methods

### 2.1. Patient cohort

This study conducted a retrospective evaluation of patients with cHCC-CC and ICC who underwent LLR at Zhejiang Provincial People’s Hospital from November 2018 to May 2023. A total of 115 patients were included in this retrospective study. Data were fully evaluated for research purposes at September 10th 2024 when human research ethics approval was obtained. This study was conducted in accordance with the Declaration of Helsinki, and the research protocol has been approved by the Ethics Committee of Zhejiang Provincial People’s Hospital (Approval NO.: ZJPPHEC 2024O(221)). Due to the retrospective nature of this study, the Ethics Committee of Zhejiang Provincial People’s Hospital has waived the requirement for patient consent for participation in the research. Among the 115 patients, 24 were confirmed to have cHCC-CC, while 91 had ICC. The exclusion criteria were: (1) patients who received any antitumor therapy prior to surgery, (2) patients with extrahepatic metastases, (3) patients undergoing recurrent liver resection, (4) presence of malignant diseases in other organs, and (5) patients with incomplete clinicopathological data or missing information.

### 2.2. Clinicopathologic features and perioperative variables

Clinicopathologic characteristics of the patients were retrospectively collected. These included gender, age at diagnosis, body mass index (BMI), American Society of Anesthesiologists (ASA) score, physical status (PS), hepatitis B virus (HBV) status, presence of cirrhosis, presence of hypertension, presence of diabetes mellitus, Child-Pugh classification, preoperative serum alanine aminotransferase (ALT) level, alanine oxalotransferase (AST) level, glutamyltranspeptidase (GGT) levels, total bilirubin (TB) levels, carcinoembryonic antigen (CEA) level, alpha-fetoprotein (AFP) level, carbohydrate antigen 19−9 (CA19−9) level, tumor size, tumor number, tumor location, TMN stage, tumor differentiation, hepatic resection type, resection margins, lymph node metastasis and microvascular invasion. The type of hepatic resection was categorized into anatomic and non-anatomic liver resection. Anatomic liver resection was defined as hepatic resection based on the anatomical structure of the liver and performed according to liver segmentation [14]. In addition, we also collected relevant perioperative variables, including operative time, intraoperative bleeding, hepatic inflow occlusion (if performed), intraoperative blood transfusion, postoperative hospitalization duration, conversions (if applicable), and postoperative complications.

### 2.3. Follow up

After LLR, all patients underwent follow-up either through clinic visits or telephone calls. For the first two years, patients were followed up every 3 months, and subsequently, every 6 months. Recurrent patients, however, were followed up monthly. Additionally, abdominal enhanced CT or MRI scans were performed every 3 months. As of May 2024, 115 patients had completed their follow-up procedures.

### 2.4. Statistical methods

Categorical variables were expressed as frequencies and percentages, and differences between groups were analyzed using the chi-squared (χ²) test or Fisher’s exact test. Continuous variables were expressed as mean ± standard deviation (SD) or median with range, and the Mann-Whitney U test was utilized to compare significant differences in these variables. Kaplan-Meier survival curves were employed to estimate prognosis.

PSM analysis was employed to mitigate selection bias [15], and cohorts were matched on propensity scores using a 1:1 ratio with a caliper width of 0.2 Due to the limited cHCC-CC cohort size (n = 24), we selected a 0.2 caliper width [16] to achieve balanced 1:1 matching without excessive sample loss from overly restrictive criteria. This approach preserved group comparability while maximizing case retention for analysis. Confounding factors incorporated into the model encompassed gender, age, BMI, ASA score, PS, cirrhosis status, HBV infection, hypertension, diabetes mellitus, Child-Pugh classification, ALT level, AST level, GGT level, TB level, tumor size, tumor number, and tumor location. The study was statistically analyzed using R software version 4.3.1. A p-value of less than 0.05 was considered statistically significant.

## 3. Results

### 3.1. Baseline characteristics

Table 1 provides a summary of the baseline characteristics of the cHCC-CC and ICC groups. A total of 115 patients were enrolled in the study cohort, comprising 24 patients with cHCC-CC) and 91 patients with ICC. The majority of patients were male (n = 74, 64.3%), and the mean age was 64.8 years. Notably, compared to ICC, cHCC-CC was more prevalent among male patients (P = 0.008). In addition to being younger, patients with cHCC-CC tended to have a higher incidence of HBV infection and cirrhosis, but these differences did not reach statistical significance (all p > 0.05). Elevated AFP levels were predominantly observed in the cHCC-CC group (45.8% vs. 2.2%, p < 0.001), while there were no significant differences in other variables between the two groups. After PSM analysis, the only significant difference in baseline characteristics between the two groups was in the elevated AFP levels (45.8% vs. 0, P < 0.001).

**Table 1:** Baseline characteristics of cHCC-CC and ICC patients.

Variables	Before PSM			After PSM		
	cHCC-CC (n = 24)	ICC (n = 91)	P	cHCC-CC (n = 24)	ICC (n = 24)	P
Gender, Male	21(87.5)	53(58.2)	0.008	21(87.5)	22(91.7)	>0.999
Age > 60 years	13(54.2)	66(72.5)	0.136	13(54.2)	16(66.7)	0.556
BMI (kg/m2)^a,b^	23.7 ± 3.8	22.8 ± 3.6	0.121	23.7 ± 3.8	23.5 ± 5.0	0.327
ASA score > 2	5(20.8)	23(25.3)	0.792	5(20.8)	6(25.0)	>0.999
Physical status ≥ 1	7(29.2)	31(34.1)	0.808	7(29.2)	9(37.5)	0.760
Cirrhosis	11(45.8)	27(29.7)	0.149	11(45.8)	9(37.5)	0.770
HBV (+)	19(79.2)	58(63.7)	0.222	19(79.2)	18(75.0)	>0.999
Diabetes mellitus	4(16.7)	12(13.2)	0.915	4(16.7)	6(25.0)	0.724
Hypertension	10(41.7)	34(37.4)	0.814	10(41.7)	10(41.7)	>0.999
Child-Pugh classification, B	1(4.2)	2(2.2)	0.508	1(4.2)	1(4.2)	>0.999
ALT > 40 IU/L	6(25.0)	16(17.6)	0.596	6(25.0)	6(25.0)	>0.999
AST > 40 IU/L	5(20.8)	16(17.6)	0.944	5(20.8)	7(29.2)	0.740
GGT > 60 IU/L	11(45.8)	43(47.3)	>0.999	11(45.8)	9(37.5)	0.770
TB > 34μmol/L	3(12.5)	6(6.6)	0.595	3(12.5)	5(20.8)	0.699
CEA > 5 µg/L	1(4.2)	18(19.8)	0.118	1(4.2)	5(20.8)	0.188
AFP > 20 µg/L	11(45.8)	2(2.2)	<0.001	11(45.8)	0(0.0)	<0.001
CA19−9 > 37U/mL	9(37.5)	49(53.8)	0.175	9(37.5)	10(41.7)	>0.999
Maximum tumor size > 5 cm	8(33.3)	28(30.8)	>0.999	8(33.3)	7(29.2)	>0.999
Multiple tumors	4(16.7)	10(11.0)	0.685	4(16.7)	4(16.7)	>0.999
Tumor location, Left hepatic lobe	6(25.0)	38(41.8)	0.233	6(25.0)	9(37.5)	0.201
Right hepatic lobe	16(66.7)	42(46.2)		16(66.7)	11(45.8)	
Median hepatic lobe	1(4.2)	9(9.9)		1(4.2)	4(16.7)	
Caudate lobe	1(4.2)	2(2.2)		1(4.2)	0(0.0)	

Data are expressed as frequencies (percentages), otherwise indicated; The chi-squared (χ²) test or Fisher’s exact test is used to compare these data differences between groups.

cHCC-CC, combined hepatocellular carcinoma and cholangiocarcinoma; ICC, intrahepatic cholangiocarcinoma; PSM, propensity score matching; BMI, body mass index; ASA, American Society of Anesthesiologists; HBV, hepatitis B virus; ALT, alanine aminotransferase; AST, alanine oxalotransferase; GGT, glutamyltranspeptidase; TB, total bilirubin; CEA, carcinoembryonic antigen; AFP, Alpha fetoprotein; CA19−9, carbohydrate antigen 19−9.

^a^Data are expressed as mean ± standard deviation.

^b^The Mann-Whitney U test is used to compare the differences in these data between groups..

#### 3.1.1. Oncologic characteristics of patients with cHCC-CC and ICC.

Table 2 presents a summary of the oncologic characteristics of the cHCC-CC and ICC groups.

**Table 2:** Oncological characteristics of laparoscopic liver resection for patients with cHCC-CC and ICC.

Variables	Before PSM			After PSM		
	cHCC-CC (n = 24)	ICC (n = 91)	P	cHCC-CC (n = 24)	ICC (n = 24)	P
AJCC-T stage,T1	10(41.7)	55(60.4)	0.111	10(41.7)	15(62.5)	0.248
TNM stage≥II	15(62.5)	45(49.5)	0.359	15(62.5)	10(41.7)	0.248
Anatomical resection	11(45.8)	73(80.2)	0.001	11(45.8)	17(70.8)	0.142
Lymph node dissection	10(41.7)	47(51.6)	0.492	10(41.7)	11(45.8)	>0.999
Number of achieved lymph nodes^a,b^	8(4-10)	6(2-35)	0.241	8(4-10)	4(2-11)	0.073
Lymph node metastasis	8(33.3)	31(34.1)	>0.999	8(33.3)	5(20.8)	0.517
Poor differentiation	6(25.0)	14(15.4)	0.433	6(25.0)	3(12.5)	0.461
Negative tumor margin	24(100.0)	87(95.6)	0.578	24(100.0)	24(100.0)	>0.999
Resection margin<1 cm	4(16.7)	21(23.1)	0.589	4(16.7)	6(25.0)	0.724
Microvascular invasion	13(54.2)	31(34.1)	0.098	13(54.2)	7(29.2)	0.142
Postoperative adjuvant chemotherapy	18(75.0)	65(71.4)	0.803	18(75.0)	19(79.2)	>0.999

Data are expressed as frequencies (percentages), otherwise indicated; The chi-squared (χ²) test or Fisher’s exact test is used to compare these data differences between groups.

cHCC-CC, combined hepatocellular carcinoma and cholangiocarcinoma; ICC, intrahepatic cholangiocarcinoma; PSM, propensity score matching.

^a^Data are expressed as median (range).

^b^The Mann-Whitney U test is used to compare the differences in these data between groups.

After PSM, the cHCC-CC group exhibited a relatively lower rate of anatomic liver resection (45.8% vs. 70.8%) and a higher prevalence of microvascular invasion (54.2% vs. 29.2%) compared to the ICC group. However, these differences did not reach statistical significance (both P > 0.05). Additionally, both the cHCC-CC and ICC groups displayed similar prevalence of T1 tumors, TNM stage>II, lymph node dissection (LND), lymph node metastasis, poor differentiation, negative tumor margin, resection margin<1 cm, and receipt of postoperative adjuvant chemotherapy. Despite these similarities, the cHCC-CC group still exhibited a tendency to have a higher number of lymph nodes achieved (8 vs. 4) compared to the ICC group, albeit without reaching statistical significance (P = 0.073).

#### 3.1.2. Perioperative outcomes.

Table 3 presents the perioperative outcomes for both the cHCC-CC and ICC groups. After PSM, the mean operative time for patients in the cHCC-CC group was 254.79 minutes, while for the ICC group, it was 265.08 minutes (P > 0.05). Compared to the ICC group, the mean intraoperative bleeding was lower in the cHCC-CC group (277.08 ml versus 402.08 ml), though this difference did not reach statistical significance (P > 0.05). There were no significant differences between the cHCC-CC and ICC groups in terms of hepatic inflow occlusion rate, intraoperative blood transfusion, mean postoperative hospitalization time, and conversion rate (P > 0.05). The rates of postoperative complications were similar in both groups (50% vs. 58.3%, P = 0.772). Patients in the cHCC-CC group had mild morbidity (Clavien-Dindo I-II), while the ICC group showed a similar trend in complication severity.

**Table 3:** Perioperative outcomes of laparoscopic liver resection for the treatment of cHCC-CC and ICC.

Variables	Before PSM			After PSM		
	cHCC-CC (n = 24)	ICC (n = 91)	P	cHCC-CC (n = 24)	ICC (n = 24)	P
Operative time (min)^a,b^	254.79 ± 113.95	280.26 ± 139.14	0.411	254.79 ± 113.95	265.08 ± 140.73	0.934
Intraoperative bleeding (ml)^a,b^	277.08 ± 234.51	354.73 ± 392.61	0.775	277.08 ± 234.51	402.08 ± 538.41	0.899
Hepatic inflow occlusion	11(45.8)	28(30.8)	0.225	11(45.8)	9(37.5)	0.770
Intraoperative blood transfusion	8(33.3)	34(37.4)	0.814	8(33.3)	6(25.0)	0.752
Postoperative hospitalization (days)^a,b^	8.00 ± 3.79	11.43 ± 7.76	0.035	8.00 ± 3.79	9.75 ± 5.99	0.302
Conversion	2(8.3)	10(11)	0.743	2(8.3)	1(4.2)	>0.999
Morbidity of complications	12(50.0)	62(68.1)	0.149	12(50.0)	14(58.3)	0.772
Clavien-Dindo			0.072			0.451
I-II	12(50.0)	49(53.8)		12(50.0)	12(50.0)	
III-IV	0(0.0)	13(11.3)		0(0.0)	2(8.3)	
Pneumonia	2(8.3)	16(17.6)	0.427	2(8.3)	4(16.7)	0.663
Hydrothorax	7(29.2)	47(51.6)	0.066	7(29.2)	8(33.3)	>0.999
Pleural effusion	4(16.7)	19(20.9)	0.863	4(16.7)	3(12.5)	>0.999
Bile leakage	1(4.2)	14(15.4)	0.267	1(4.2)	4(16.7)	0.345

Data are expressed as frequencies (percentages), otherwise indicated; The chi-squared (χ²) test or Fisher’s exact test is used to compare these data differences between groups.

cHCC-CC, combined hepatocellular carcinoma and cholangiocarcinoma; ICC, intrahepatic cholangiocarcinoma; PSM, propensity score matching.

^a^Data are expressed as mean ± standard deviation.

^b^The Mann-Whitney U test is used to compare the differences in these data between groups.

#### 3.1.3. Overall survival and recurrence-free survival.

**Fig 1:**
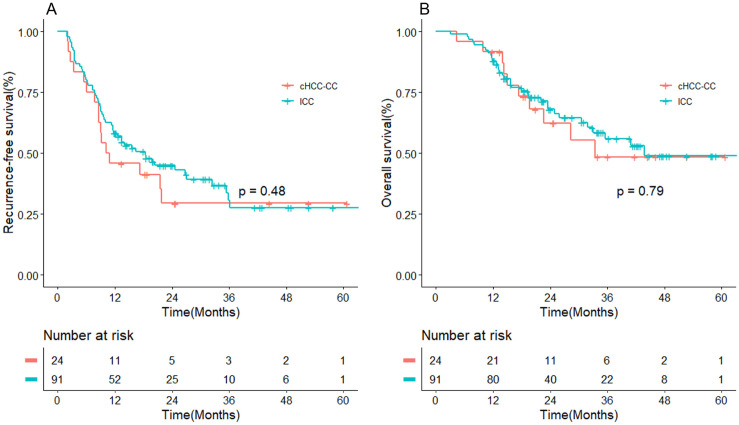
Comparison of the overall survival rate and recurrence-free survival rate curves between patients with cHCC-CC and ICC before propensity score matching. (A) Recurrence-free survival rate; (B) Overall survival rate.

The median follow-up for the entire cohort was 34.0 months. During this follow-up period, 16 (66.7%) patients in the cHCC-CC group experienced recurrence, and 10 (41.7%) patients died. The 1-year, 2-year, and 3-year recurrence-free survival (RFS) estimates for the cHCC-CC group were 46%, 29%, and 29%, respectively. The corresponding 1-year, 2-year, and 3-year overall survival (OS) estimates were 92%, 69%, and 49%, respectively. In comparison, in the ICC group, 55 (60.4%) patients experienced recurrence and 34 (37.4%) patients died. The estimated 1-year, 2-year, and 3-year RFS for the ICC group was 58%, 45%, and 31%, respectively. The corresponding 1-year, 2-year, and 3-year OS estimates were 89%, 68%, and 56%, respectively. The Kaplan-Meier curves demonstrated no statistically significant difference in RFS and OS between the cHCC-CC and ICC groups (P > 0.05) (Fig 1).

**Fig 2:**
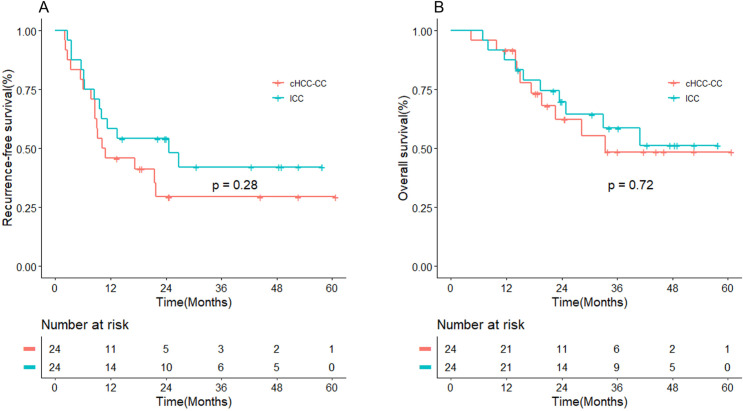
Comparison of the overall survival rate and recurrence-free survival rate curves between patients with cHCC-CC and ICC after propensity score matching. (A) Recurrence-free survival rate; (B) Overall survival rate.

After PSM, the estimated RFS in the cHCC-CC group at 1, 2, and 3 years was 46%, 29%, and 29%, respectively. The corresponding estimated OS was 92%, 62%, and 49%, respectively. In comparison, the estimated RFS in the ICC group at 1, 2, and 3 years was 58%, 54%, and 42%, respectively, and the estimated OS was 88%, 70%, and 59%, respectively. The Kaplan-Meier curves showed that RFS and OS were similar in the two groups (P > 0.05) (Fig 2).

## 4. Discussion

cHCC-CC is a rare primary hepatic malignancy that rarely occurs in clinical practice. Currently, there are no standardized treatment guidelines for its management. Some studies have indicated that it shares more similarities with ICC [4,17]. The 8th edition of the American Joint Committee on Cancer (AJCC) staging manual employs the same staging system for both cHCC-CC and ICC [18]. LLR has evolved as a highly sophisticated treatment option for ICC, supported by extensive literature showing its safety and effectiveness in managing both small isolated tumors and large or multiple tumors [10,19]. However, there is limited data regarding the safety of laparoscopic techniques in treating patients with cHCC-CC. A total of 115 patients were included in our study, with 24 patients finally diagnosed with cHCC-CC and 91 patients ICC. Our study findings indicate that LLR is equally safe and effective in the treatment of cHCC-CC. Additionally (data not tabulated), of the 24 cHCC-CC patients, 16 (66.7%) developed recurrent disease, with initial recurrence being intrahepatic in 11 cases (45.8%) and extrahepatic in 5 (20.8%). Among 91 ICC patients, 55 cases (60.4%) developed recurrent disease, predominantly intrahepatic (44 cases, 48.4%) compared to extrahepatic recurrence (11 cases, 12.1%). The study cohort demonstrated comparable rates of postoperative adjuvant chemotherapy administration between groups, with 18 cHCC-CC patients (75.0%) and 65 ICC patients (71.4%) receiving treatment (p = 0.803). Furthermore, we observed that the perioperative efficacy and long-term prognosis of patients with cHCC-CC treated using laparoscopic techniques are comparable to those of patients with ICC. Moreover, to date, there has been no relevant literature comparing the perioperative outcomes between LLR for cHCC-CC and ICC. Our study revealed no significant differences between the two groups in terms of operative time, intraoperative blood loss, conversion rate, LND rate, length of postoperative stay, and postoperative complication rate. There were no life-threatening complications and no deaths.

The clinicopathologic characteristics of patients with cHCC-CC and ICC are not entirely analogous, exhibiting differences in certain variables. Specifically, compared to ICC, cHCC-CC has a higher incidence among male patients, tends to occur in a younger population, and has a higher prevalence of HBV and cirrhosis. These findings align with previous reports in the literature [20–22]. AFP and CA 19−9 are potential effective biomarkers for cHCC-CC. In this article, there was a significant difference in AFP levels between the cHCC-CC group and the ICC group, with 45.8% of patients with cHCC-CC having abnormally elevated AFP levels and 37.5% having abnormally elevated CA19−9 levels. These findings are consistent with previous reports [23,24]. Therefore, when both AFP and CA19−9 are elevated simultaneously, or when the elevation of tumor markers does not align with imaging findings, it suggests the possible existence of this specific tumor type, namely cHCC-CC [25,26].

One of the topics of interest in the laparoscopic treatment of cHCC-CC is whether LND is necessary. Currently, for patients with ICC, numerous studies recommend routine hilar LND and resection of at least 6 lymph nodes, as this can provide accurate staging of ICC [27,28]. However, whether LND actually improves prognosis remains a subject of debate [29,30]. There are fewer reports on whether patients with cHCC-CC should also undergo routine LND. In our study, 41.7% of cHCC-CC patients underwent regional LND, and this percentage did not significantly differ from that of the ICC group. Moreover, the lymph node positivity rates were similar in both groups. Notably, performing additional LND may increase the duration of the operation and cause more damage. However, necessary LND is essential for patients with lymph node positivity, to achieve accurate disease staging, and for guiding subsequent follow-up treatment.

Another important consideration is the use of anatomical resection. Since cHCC-CC exhibits both HCC and ICC components, the tumor can metastasize intrahepatically through vascular structures and spread through the biliary tree and lymphatic system. As a result, anatomical resection may be a more favorable surgical option for patients with cHCC-CC. It has been reported in the literature that 5-year OS and RFS rates are significantly higher for patients with cHCC-CC who undergo anatomical resection compared to those receiving non-anatomical resection [14]. Our analysis revealed striking differences in preoperative diagnostic accuracy between the two groups, with only 5 cases (20.8%) of cHCC-CC being correctly diagnosed compared to 53 cases (58.2%) of ICC through imaging (data not tabulated). This substantial diagnostic disparity (20.8% vs 58.2%) likely impacts surgical decision-making, particularly in selecting anatomical resection strategies. Interestingly, while not statistically significant (P > 0.05), we observed that cHCC-CC patients underwent anatomical resection at relatively lower rates than ICC patients. This pattern may be explained by the well-documented challenges in radiologically diagnosing cHCC-CC preoperatively, which frequently leads to misdiagnosis as HCC. Consequently, many cHCC-CC patients received surgical approaches (either anatomical or non-anatomical resections) originally intended for HCC management, potentially explaining the observed differences in resection strategies between the two tumor types. Future research is worth exploring the impact of anatomical and non-anatomical resection on cHCC-CC.

As indicated in previous studies, the long-term prognosis of patients with cHCC-CC is more akin to that of ICC [21,31]. Our study found a median OS of 33.4 months in the cHCC-CC group after LLR, which aligns closely with the median OS of 33.0 months reported in a prior study [32].

Interestingly, it was found that in matched cohorts after open surgery, cHCC-CC showed comparable OS to ICC (p = 0.06), while demonstrating better disease-free survival than ICC (p < 0.05) [33]. In our study, survival analysis revealed similar OS and RFS between the cHCC-CC and ICC groups. As mentioned, recurrence occurred in 16/24 (66.7%) cHCC-CC patients (45.8% intrahepatic, 20.8% extrahepatic) versus 55/91 (60.4%) ICC patients (48.4% vs 12.1%). These findings carry significant surgical implications. Given cHCC-CC’s well-documented propensity for biliary tract and lymphatic system dissemination, our data suggest that aggressive surgical approaches—including systematic LND and extended hepatectomy—may be particularly warranted to address both the predominant intrahepatic recurrence pattern and potential micrometastatic disease.

There are indeed several limitations to this study. First, as a single-center retrospective study, it is inevitable that there is a risk of patient selection bias. Second, the relatively short median follow-up period constrains our ability to analyze long-term outcomes. Third, although the lymph node positivity rates between the two groups (33.3% versus 34.1%, P > 0.05) were similar, the LND rates (41.7% versus 51.6%, P > 0.05) under laparoscopy were not high in both groups in this study, which may affect the interpretation of surgical outcomes. Future research is worthwhile to investigate the impact of LND on surgical results. Fourth, there were only 24 patients in the cHCC-CC group, which is not a large enough sample size to provide conclusive evidence. The modest cHCC-CC sample size (n = 24) may affect the PSM robustness. Hence, future studies should employ prospective, multicenter designs with larger sample sizes to strengthen our findings and delve deeper into the subject matter.

## 5. Conclusion

The perioperative outcomes and long-term prognosis of LLR for patients with cHCC-CC are comparable to those observed in patients with ICC. Future studies with better designs and larger sample sizes are warranted to strengthen our research findings.

## References

[1] Beaufrère A, Calderaro J, Paradis V. Combined hepatocellular-cholangiocarcinoma: an update. J Hepatol. 2021;74(5):1212–24. doi: 10.1016/j.jhep.2021.01.035 33545267

[2] Nagtegaal ID, Odze RD, Klimstra D, Paradis V, Rugge M, Schirmacher P, et al. The 2019 WHO classification of tumours of the digestive system. Histopathology. 2020;76(2):182–8. doi: 10.1111/his.13975 31433515 PMC7003895

[3] Ramai D, Ofosu A, Lai JK, Reddy M, Adler DG. Combined hepatocellular cholangiocarcinoma: a population-based retrospective study. Am J Gastroenterol. 2019;114(9):1496–501. doi: 10.14309/ajg.0000000000000326 31335362

[4] Liu D, Heij LR, Czigany Z, Dahl E, Dulk M den, Lang SA, et al. The prognostic value of neutrophil-to-lymphocyte ratio in cholangiocarcinoma: a systematic review and meta-analysis. Sci Rep. 2022;12(1):12691. doi: 10.1038/s41598-022-16727-w 35879385 PMC9314341

[5] Li Y, He D, Lu Z-J, Gu X-F, Liu X-Y, Chen M, et al. Clinicopathological characteristics and prognosis of combined hepatocellular cholangiocarcinoma. BMC Cancer. 2024;24(1):1232. doi: 10.1186/s12885-024-12970-8 39375615 PMC11457400

[6] Spolverato G, Bagante F, Tsilimigras D, Ejaz A, Cloyd J, Pawlik TM. Management and outcomes among patients with mixed hepatocholangiocellular carcinoma: a population-based analysis. J Surg Oncol. 2019;119(3):278–87. doi: 10.1002/jso.25331 30554420

[7] Renzulli M, Ramai D, Singh J, Sinha S, Brandi N, Ierardi AM, et al. Locoregional treatments in cholangiocarcinoma and combined hepatocellular cholangiocarcinoma. Cancers (Basel). 2021;13(13):3336. doi: 10.3390/cancers13133336 34283065 PMC8268054

[8] Yamashita Y-I, Aishima S, Nakao Y, Yoshizumi T, Nagano H, Kuroki T, et al. Clinicopathological characteristics of combined hepatocellular cholangiocarcinoma from the viewpoint of patient prognosis after hepatic resection: high rate of early recurrence and its predictors. Hepatol Res. 2020;50(7):863–70. doi: 10.1111/hepr.13507 32335986

[9] Pery R, Gudmundsdottir H, Nagorney DM, Pencovich N, Smoot RL, Thiels CA, et al. Laparoscopic versus open liver resections for intrahepatic cholangiocarcinoma and gallbladder cancer: the Mayo clinic experience. HPB. 2023;25(3):339–46. doi: 10.1016/j.hpb.2022.12.006 36707278

[10] Wei F, Lu C, Cai L, Yu H, Liang X, Cai X. Can laparoscopic liver resection provide a favorable option for patients with large or multiple intrahepatic cholangiocarcinomas? Surg Endosc. 2017;31(9):3646–55. doi: 10.1007/s00464-016-5399-3 28032221

[11] Patrone R, Izzo F, Palaia R, Granata V, Nasti G, Ottaiano A, et al. Minimally invasive surgical treatment of intrahepatic cholangiocarcinoma: a systematic review. World J Gastrointest Oncol. 2021;13(12):2203–15. doi: 10.4251/wjgo.v13.i12.2203 35070052 PMC8713325

[12] Regmi P, Hu H-J, Paudyal P, Liu F, Ma W-J, Yin C-H, et al. Is laparoscopic liver resection safe for intrahepatic cholangiocarcinoma? A meta-analysis. Eur J Surg Oncol. 2021;47(5):979–89. doi: 10.1016/j.ejso.2020.11.310 33339638

[13] Ziogas IA, Esagian SM, Giannis D, Hayat MH, Kosmidis D, Matsuoka LK, et al. Laparoscopic versus open hepatectomy for intrahepatic cholangiocarcinoma: an individual patient data survival meta-analysis. Am J Surg. 2021;222(4):731–8. doi: 10.1016/j.amjsurg.2021.03.052 33840443

[14] Wang W-Q, Li J, Liang B-Y, Lv X, Zhu R-H, Wang J-L, et al. Anatomical liver resection improves surgical outcomes for combined hepatocellular-cholangiocarcinoma: a propensity score matched study. Front Oncol. 2022;12:980736. doi: 10.3389/fonc.2022.980736 36059669 PMC9433922

[15] Kane LT, Fang T, Galetta MS, Goyal DKC, Nicholson KJ, Kepler CK, et al. Propensity score matching. Clin Spine Surg. 2020;33(3):120–2. doi: 10.1097/bsd.0000000000000932 31913173

[16] Austin PC. Optimal caliper widths for propensity-score matching when estimating differences in means and differences in proportions in observational studies. Pharm Stat. 2011;10(2):150–61. doi: 10.1002/pst.433 20925139 PMC3120982

[17] Brunt E, Aishima S, Clavien P, Fowler K, Goodman Z, Gores G, et al. cHCC‐CCA. Hepatology. 2018;68(1):113–26. doi: 10.1002/hep.29789 29360137 PMC6340292

[18] Amin MB, Greene FL, Edge SB, Compton CC, Gershenwald JE, Brookland RK, et al. The Eighth Edition AJCC cancer staging manual: continuing to build a bridge from a population-based to a more “personalized” approach to cancer staging. CA Cancer J Clin. 2017;67(2):93–9. doi: 10.3322/caac.21388 28094848

[19] Li H, Wang Q, Yang Z, Zhu F, Xiang Z, Long Z, et al. Laparoscopic versus open hepatectomy for intrahepatic cholangiocarcinoma: systematic review and meta-analysis of propensity score-matched studies. Europ J Surg Oncol. 2023;49(4):700–8. doi: 10.1016/j.ejso.2023.02.010 36842897

[20] Yang Z, Shi G. Survival outcomes of combined hepatocellular-cholangiocarcinoma compared with intrahepatic cholangiocarcinoma: a SEER population-based cohort study. Cancer Med. 2022;11(3):692–704. doi: 10.1002/cam4.4474 34862762 PMC8817088

[21] Lin C-W, Wu T-C, Lin H-Y, Hung C-M, Hsieh P-M, Yeh J-H, et al. Clinical features and outcomes of combined hepatocellular carcinoma and cholangiocarcinoma versus hepatocellular carcinoma versus cholangiocarcinoma after surgical resection: a propensity score matching analysis. BMC Gastroenterol. 2021;21(1):20. doi: 10.1186/s12876-020-01586-4 33413162 PMC7788698

[22] Schizas D, Mastoraki A, Routsi E, Papapanou M, Tsapralis D, Vassiliu P, et al. Combined hepatocellular-cholangiocarcinoma: an update on epidemiology, classification, diagnosis and management. Hepatobiliary Pancreat Dis Int. 2020;19(6):515–23. doi: 10.1016/j.hbpd.2020.07.004 32753331

[23] Li R, Yang D, Tang C-L, Cai P, Ma K-S, Ding S-Y, et al. Combined hepatocellular carcinoma and cholangiocarcinoma (biphenotypic) tumors: clinical characteristics, imaging features of contrast-enhanced ultrasound and computed tomography. BMC Cancer. 2016;16:158. doi: 10.1186/s12885-016-2156-x 26917546 PMC4768404

[24] Zhang G, Chen B-W, Yang X-B, Wang H-Y, Yang X, Xie F-C, et al. Prognostic analysis of patients with combined hepatocellular-cholangiocarcinoma after radical resection: a retrospective multicenter cohort study. World J Gastroenterol. 2022;28(41):5968–81. doi: 10.3748/wjg.v28.i41.5968 36405111 PMC9669829

[25] Chen J, Li Y, Yu G. Diagnostic value of serum biomarkers in combined hepatocelluar-cholangiocarcinoma. J Coll Physicians Surg Pak. 2020;30(3):263–7. doi: 10.29271/jcpsp.2020.03.263 32169133

[26] Ye L, Schneider JS, Ben Khaled N, Schirmacher P, Seifert C, Frey L, et al. Combined hepatocellular-cholangiocarcinoma: biology, diagnosis, and management. Liver Cancer. 2023;13(1):6–28. doi: 10.1159/000530700 38344449 PMC10857821

[27] Benson AB, D’Angelica MI, Abbott DE, Anaya DA, Anders R, Are C, et al. Hepatobiliary cancers, version 2.2021, NCCN clinical practice guidelines in oncology. J Natl Compr Canc Netw. 2021;19(5):541–65. doi: 10.6004/jnccn.2021.0022 34030131

[28] Li F, Jiang Y, Jiang L, Li Q, Yan X, Huang S, et al. Effect of lymph node resection on prognosis of resectable intrahepatic cholangiocarcinoma: a systematic review and meta-analysis. Front Oncol. 2022;12:957792. doi: 10.3389/fonc.2022.957792 36237310 PMC9552707

[29] Umeda Y, Mitsuhashi T, Kojima T, Satoh D, Sui K, Endo Y, et al. Impact of lymph node dissection on clinical outcomes of intrahepatic cholangiocarcinoma: Inverse probability of treatment weighting with survival analysis. J Hepatobiliary Pancreat Sci. 2022;29(2):217–29. doi: 10.1002/jhbp.1038 34473411 PMC9291593

[30] Vitale A, Moustafa M, Spolverato G, Gani F, Cillo U, Pawlik TM. Defining the possible therapeutic benefit of lymphadenectomy among patients undergoing hepatic resection for intrahepatic cholangiocarcinoma. J Surg Oncol. 2016;113(6):685–91. doi: 10.1002/jso.24213 26936676

[31] Yoon Y-I, Hwang S, Lee Y-J, Kim K-H, Ahn C-S, Moon D-B, et al. Postresection outcomes of combined hepatocellular carcinoma-cholangiocarcinoma, hepatocellular carcinoma and intrahepatic cholangiocarcinoma. J Gastrointest Surg. 2016;20(2):411–20. doi: 10.1007/s11605-015-3045-3 26628072

[32] Song D-J, Zhu K, Tan J-P, Cai J-B, Lv M-Z, Hu J, et al. Perioperative and oncologic outcomes of laparoscopic versus open liver resection for combined hepatocellular-cholangiocarcinoma: a propensity score matching analysis. Surg Endosc. 2023;37(2):967–76. doi: 10.1007/s00464-022-09579-y 36076103

[33] Tang Y, Wang L, Teng F, Zhang T, Zhao Y, Chen Z. The clinical characteristics and prognostic factors of combined hepatocellular carcinoma and cholangiocarcinoma, hepatocellular carcinoma and intrahepatic cholangiocarcinoma after surgical resection: a propensity score matching analysis. Int J Med Sci. 2021;18(1):187–98. doi: 10.7150/ijms.50883 33390787 PMC7738961

